# Perceptions of barriers to and facilitators of exercise rehabilitation in adults with lung transplantation: a qualitative study in China

**DOI:** 10.1186/s12890-024-02882-5

**Published:** 2024-02-01

**Authors:** Hui Yang, Saisai Liu, Jingru Chen, Yaxin Qiao, Chengcheng Wang, Wenping Zhang, Li Wei, Ruiyun Chen

**Affiliations:** 1 Department of Thoracic surgery, Henan Key Laboratory for Nursing, Henan Provincial People’s Hospital, People’s Hospital of Zhengzhou University, Henan University People’s Hospital, No.7 Weiwu Road, Jinshui District, Zhengzhou, 450003 Henan Province People’s Republic of China; 2 Institute of Nursing and Health, Henan University, Kaifeng, 475004 Henan China; 3 Department of Thoracic surgery, Henan Provincial People’s Hospital, People’s Hospital of Zhengzhou University, Henan University People’s Hospital, Zhengzhou, 450003 Henan China

**Keywords:** Barriers, Facilitators, Exercise rehabilitation, Lung transplantation, Qualitative research

## Abstract

**Background:**

Exercise is crucial for pulmonary rehabilitation and improving the prognosis of lung transplantation (LTx) patients. However, many LTx patients in China have low exercise tolerance and compliance, and the reasons behind these challenges have not been fully elucidated. Therefore, this qualitative research aims to identify the barriers to and facilitators of exercise rehabilitation in LTx patients.

**Methods:**

From January to July 2023, 15 stable LTx patients were recruited and participated in in-depth, semi-structured, face-to-face interviews at Henan Provincial People’s Hospital. The interview transcripts were analyzed using the COM-B model and the Theoretical Domains Framework (TDF).

**Results:**

Six general themes including 19 barriers and 14 facilitators for the exercise rehabilitation of LTx patients were identified based on the COM-B model and TDF. The barriers to exercise included physical limitations, insufficient exercise endurance, lack of knowledge, and lack of motivation. The facilitators of exercise included motivation, self-efficacy, perceived significance of exercise rehabilitation, and social support.

**Conclusion:**

The study offers detailed insight into the development and implementation of exercise rehabilitation intervention strategies for LTx patients. By combining COM-B model and TDF, the study provides strong evidence that active behavior change strategies are required for LTx patients to promote their participation in exercise rehabilitation. Professional support, pulmonary rehabilitation training, behavior change technology, and digital health tools are essential for strengthening the evidence system for reporting exercise efficacy and effectiveness.

## Introduction

Lung transplantation (LTx) is the sole treatment option for end-stage lung diseases [1, 2], and the number of patients on LTx worldwide is steadily increasing [3]. In China, a total of 1053 LTx cases were recorded in the Lung Transplantation Registry between 2015 and 2018. The postoperative survival rates at 1-, 3-, and 5-year were 70.11, 61.16, and 54% respectively, showing a gradual decline [4].

Exercise plays a crucial role in pulmonary rehabilitation, especially in improving the prognosis of LTx patients [5, 6]. Exercise is defined as planned, structured, repetitive, and purposeful activities aimed at enhancing or maintaining overall health [7]. Numerous studies have demonstrated that exercise rehabilitation can alleviate secondary chronic transplant-related conditions like hypertension, metabolic syndrome, and osteoporosis, as well as improve health-related quality of life [8–10]. In addition, exercise is a specific category of physical activity (PA), which encompasses any movement generated by skeletal muscles that expends energy [7]. Engaging in regular PA is immensely beneficial, particularly for patients recuperating from LTx. It not only enhances exercise capacity but also decreases the risk of developing complications such as osteoporosis, muscle dysfunction, metabolic issues, and cardiovascular disorders [11]. Moreover, an active lifestyle post-transplantation is associated with elevated survival rates and a reduced incidence of secondary injuries in patients [12]. In their evaluation of PA levels, Gustaw T et al. [12] utilized the Physical Activity Scale for the Elderly (PASE) to establish three distinct classifications of activity: limited (0–50 points), low (50.1–200 points), and high (> 200.1 points). However, despite the evidence supporting the benefits of PA and structured exercise training in solid organ transplant recipients, many patients still have low levels of PA for several years after surgery [12]. In fact, almost 90% of LTx patients are considered inactive [13], with exercise adherence rates about 50%, and their exercise capability declined 40–60% within 2 years [14, 15]. Nonetheless, the causes of low exercise tolerance and compliance in LTx patients are not fully elucidated.

Currently, some studies abroad have surveyed the exercise aspects of post-organ transplant patients, and found that these patients encountered various barriers to exercise rehabilitation, including physical factors (e.g., lack of energy, medication side effects), psychological factors (e.g., low self-efficacy), and social factors (e.g., lack of social support) [8, 16–19]. Promoting factors for exercise in these patients included digital health interventions such as smartphone apps, a desire to live long and healthy, renewed physical capabilities, access to appropriate fitness guidelines and facilities, motivation, understanding the consequences of being active or inactive, adhering to routines or habits, setting goals and prioritizing them, taking responsibility for the transplanted organ, group exercise, and peer support [18, 20–22]. However, studies on LTx patients in China were limited and the outcomes were unsatisfactory [23]. Li et al. [24] conducted a cross-sectional survey on 169 LTx recipients’ rehabilitation status during out-of-hospital exercise. The results highlighted the association between low exercise compliance and factors such as age, postoperative time, surgical method, perceived severity and benefits, self-efficacy, and family support. Given differences in patients’ physical fitness and lifestyle habits between Chinese patients and foreign patients, it is crucial to explore the barriers to and facilitators of exercise rehabilitation in China as a basis for targeted interventions.

**Fig. 1:**
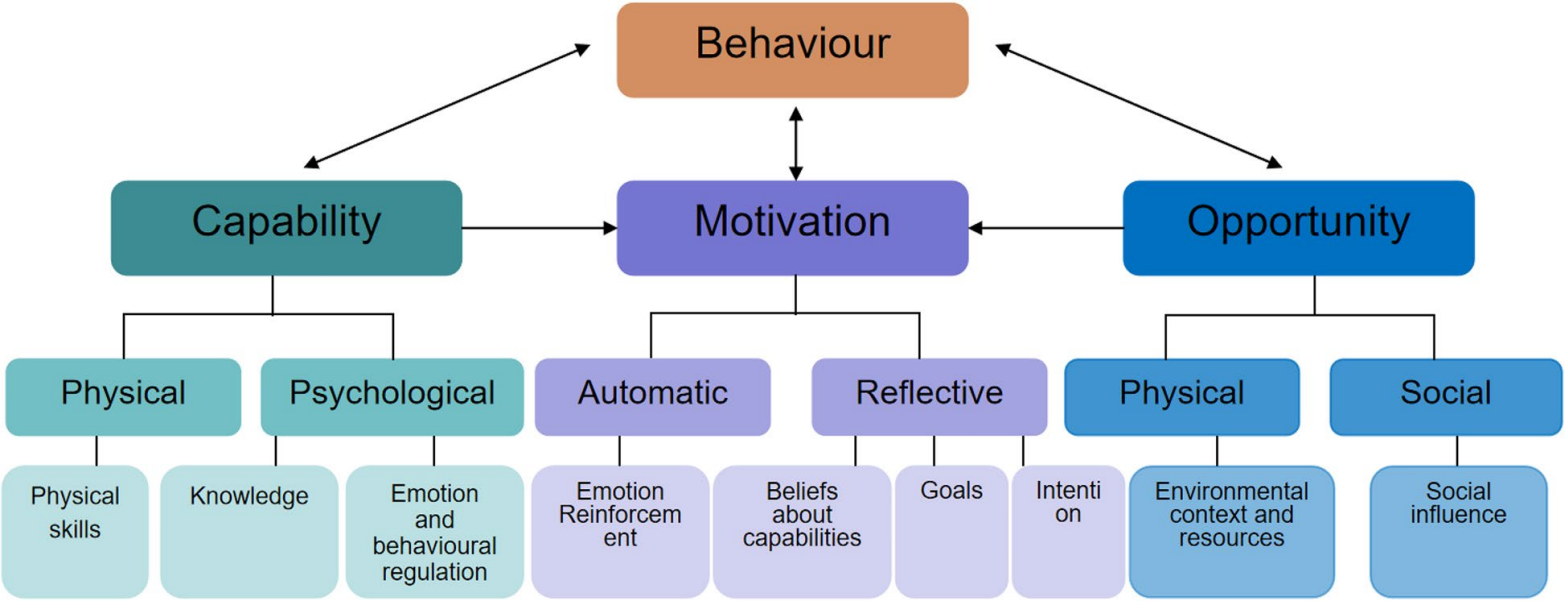
Theoretical framework

In order to improve the exercise status of LTx patients in China, we attempted to summarize the factors affecting exercise behavior based on the behavior change model proposed by Michie et al. [25]—the “Capacity, Opportunity, Motivation, Behavior (COM-B)” model. In this model, capacity (psychological and physiological abilities, such as knowledge and skills), opportunity (physical and social environments, such as environmental resources and social influences), and motivation factors (reflexive and spontaneous motivations, such as self-efficacy) interact with each other and influence behavior. Ability and opportunity factors can influence behavior directly, or indirectly through motivation factors. To identify more detailed influencing factors, this study incorporated the theoretical domains framework (TDF). Michie et al. [26] systematically screened 33 behavior change theories and then integrated them into TDF to find the most comprehensive behavior change theory to guide the behavior intervention research. The TDF encompasses factors at individual, organizational, and societal levels, facilitating researchers to explore the determinants of behavior from diverse perspectives [27]. These two theories have been successfully applied to behavior change intervention strategies in healthcare systems [26]. The TDF can support the COM-B model in identifying the specific and comprehensive influencing factors for target behaviors, with each theoretical domain related to a specific part of the COM-B model. Therefore, this study combined COM-B model with TDF and designed an interview outline to deeply and comprehensively explore the influencing factors of exercise behavior in Chinese LTx patients (Fig. 1).

### Purpose

The purpose of this qualitative study was to explore the barriers and facilitators of exercise rehabilitation in LTx patients based on the COM-B theoretical model and TDF.

## Methods

### Design

The study conducted interviews with patients who had maintained a stable condition after LTx. This qualitative study was approved by the Medical Ethics Committee of Henan Provincial People’s Hospital (IRB #: 2022120). All interviewees signed the informed consent form.

A qualitative study was undertaken to elucidate the barriers and facilitators of exercise among stabilized recipients following LTx. Between January and July 2023, in-depth, semi-structured, and face-to-face interviews were conducted with 15 stabilized LTx patients [28].

Based on previous literature research, an open-ended, semi-structured interview outline was formulated, covering four areas: 1) Exercise situation; 2) Promoting factors of exercise; 3) Obstacle factors of exercise; 4) What kind of guidance do you want from medical staff. Before the interviews, rapport was established between the researchers and participants, and the purpose of the study was explained. The collected data were strictly used for this study and maintained anonymity and confidentiality. Participants had the right to discontinue at any point. This study followed the Standards for Reporting Qualitative Research [29].

### Participants and recruitment

LTx patients were recruited from the thoracic surgery wards of Henan Provincial People’s Hospital. The determination of the sample size was anchored upon achieving data saturation, which, rather than being ascertained during the interviews, was concluded prior to the completion of the analysis phase. Our approach hinged on the deductive method, grounded in theoretical frameworks, with ‘deductive topic saturation’ serving as our benchmark. This was established when the points of view from the patients encompassed all theoretical topics at least three times [30, 31]. Drawing on previous research experience and the principles of deduction, we initially posited a minimum of 12 cases for the early phase of our study. However, according to our analysis, the final sample size was set at 15 cases [28, 30, 31]. Inclusion criteria were as follows: patients who had undergone LTx and were in a stable condition were included, including those who had been discharged to continue exercising at home and who regularly returned to the hospital for follow-up visits. On the other hand, the exclusion criteria were as follows: the requirement for mechanical ventilation, contraindications for PA, the presence of severe heart, brain, or kidney conditions, and patients with verbal communication difficulties. Additionally, individuals who withdrew from the study for various reasons at any point were also excluded. General patient information was obtained by retrieving medical records.

### Data collection

One-on-one interviews were conducted at the meeting room in Hospital, where is a relatively quiet, comfortable, and secluded place. Each interview lasted 30–60 minutes and was recorded through audio and video recorder with the consent of the patient. Two nurses were involved in the interviews, with one primarily asking questions and the other taking detailed notes. The camera and voice recorder recorded content included both the spoken words and nonverbal actions of the patients. The accuracy of the transcripts was ensured by reading them while simultaneously listening to the recorded files. Nvivo 11 (QSR International, Chadstone, VIC, Australia). Software was used for transcribing, coding, and extracting themes from the interview recordings. The final themes were translated into English, and selected quotes were back-translated to ensure accuracy.

### Data analysis

The data collected was subjected to a six-step thematic analysis procedure [32], combined with a deductive methodology. 1) Acquainting oneself with the data and making relevant notes. 2) Initial codes were generated by two researchers who independently coded the interview content twice. They identified both promoting and inhibiting factors and deductively coded these factors into a theoretical behavioral change framework of TDF. 3) The initial codes were organized into possible themes within the context of the COM-B model. 4) Potential themes were reviewed. Some preliminary codes, identified in our previous analyses, may not fit into pre-established themes and therefore were adjusted. 5) Themes were precisely defined and named. 6) A comprehensive report was produced. Analysis of the interview content revealed that integrating the interview themes with the COM-B model provided a comprehensive representation. Moreover, the TDF played a crucial role in refining the identification of target behaviors within the COM-B model. Therefore, the COM-B model was integrated with the TDF to present the identified themes.

## Results

**Fig. 2:**
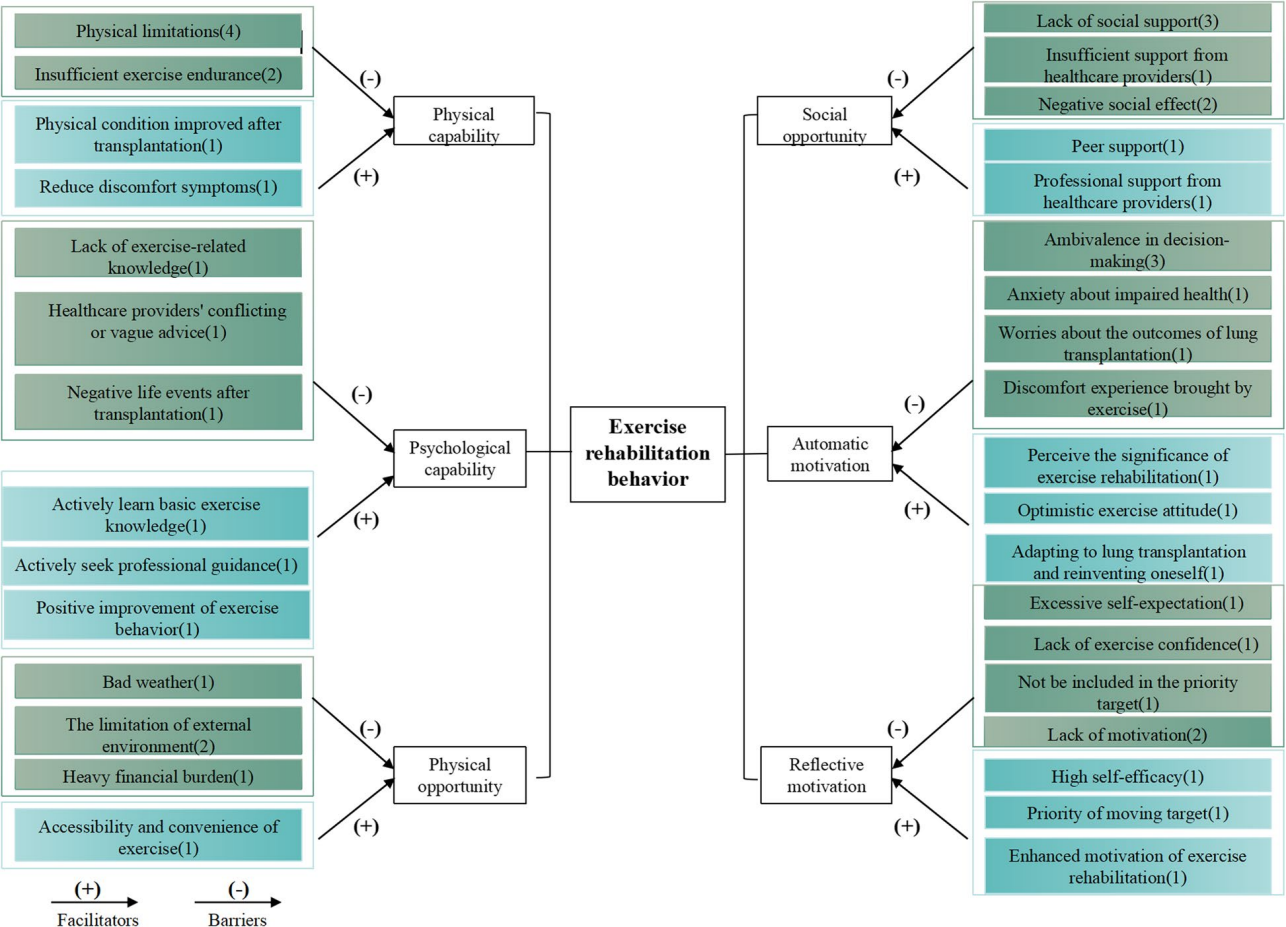
Influencing factors of exercise rehabilitation in LTx patients

A total of 15 participants were interviewed. Table 1 shows the characteristics of the participants. From our analysis, guided by the COM-B theory and TDF, six overarching themes and nine domains for the exercise rehabilitation of LTx patients were discerned. These themes included physical capability, psychological capability, physical opportunity, social opportunity, automatic motivation, and reflective motivation. Additionally, there were 19 identified barriers and 14 facilitators that influence these patients’ exercise rehabilitation journey, all in accordance with the TDF embedded in the COM-B model, as depicted in Fig. 2 and Table 2.

**Table 1:** Characteristics of participants

Participant ID	Genger	Ages (years)	BMI (kg/m^2^)	Highest level of education	Employment	Annual household income	Medical insurance	Transplant time (months)	Complications	Current state
1	Male	24	17.48	Elementary school	Unemployed	Middle-income	Resident medical insurance	0.3	–	In hospital
2	Female	56	23.32	High school	Retired	High-income	Employee medical insurance	0.5	Hypertension	At home
3	Male	69	24.80	High school	Unemployed	Middle-income	NRCMS	23	Hypertension, diabetes	At home
4	Male	33	23.65	Junior school	Employed	High-income	NRCMS	3	–	In hospital
5	Male	55	23.30	Junior school	Unemployed	High-income	NRCMS	15	–	At home
6	Male	56	14.86	High school	Retired	High-income	Employee medical insurance	6	Diabetes	In hospital
7	Male	68	27.76	High school	Unemployed	Middle-income	Resident medical insurance	0.1	–	In hospital
8	Female	51	23.14	Elementary school	Unemployed	Low-income	NRCMS	0.5	Hypertension	At home
9	Male	59	22.41	High school	Retired	Middle-income	Employee medical insurance	43	–	At home
10	Male	75	24.16	Elementary school	Unemployed	Middle-income	Resident medical insurance	2	Hypertension	In hospital
11	Male	45	25.21	High school	Unemployed	Middle-income	NRCMS	13	–	In hospital
12	Male	50	21.34	College/university	Employment	High-income	Employee medical insurance	14	–	In hospital
13	Female	49	25.30	High school	Employment	Middle-income	Employee medical insurance	31	–	At home
14	Male	57	27.31	Junior school	Unemployed	Low-income	NRCMS	0.5	–	In hospital
15	Male	61	24.45	College/university	Unemployed	Middle-income	Commercial insurance	2	Coronary heart disease	In hospital

Abbreviate: *NRCMS* New rural cooperative medical system

**Table 2:** Identified barriers and facilitators to exercise rehabilitation classified according to TDF embedded in COM-B

COM-B	TDF	Barriers	Facilitators
Physical capability	Physical skills	• Physical limitations • Insufficient exercise endurance	• Physical condition improved after transplantation • Reduce discomfort symptoms
Psychological capability	Knowledge Emotion and behavioural regulation	• Lack of exercise-related knowledge • Healthcare providers’ conflicting or vague advice • Negative life events after transplantation	• Actively learn basic exercise knowledge • Actively seek professional guidance • Positive improvement of exercise behavior
Physical opportunity	Environmental context and resources	• Bad weather • The limitation of external environment • Heavy financial burden	• Accessibility and convenience of exercise
Social opportunity	Social influence	• Lack of social support • Insufficient support from healthcare providers • Negative social effect	• Peer support • Professional support from healthcare providers
Automatic motivation	Emotion Reinforcement	• Ambivalence in decision-making • Anxiety about impaired health • Worries about the outcomes of LTx • Discomfort experience brought by exercise	• Perceive the significance of exercise rehabilitation • Optimistic exercise attitude • Adapting to LTx and reinventing oneself
Reflective motivation	Beliefs about capabilities Goals Intention	• Excessive self-expectation • Lack of exercise confidence • Not be included in the priority target • Lack of motivation	• High self-efficacy • Priority of moving target • Enhanced motivation of exercise rehabilitation

### Physical capability

#### Barriers

##### Physical limitations (pay, pipeline, drug side effects, comorbidities)

Ten participants (66.67%) reported that physical limitations were a bad rehabilitation experience that hindered the participants’ exercise rehabilitation and brought physical discomfort, including pain, pipelines, drug side effects, and comorbidities.

“I feel particularly painful after the operation. The wound is so big that I can’t sleep, let alone exercise.” (P1, 24 years).

Five participants (33.33%) pointed out that indwelling tubes after LTx, such as thoracic drainage tube, urinary catheter, and central venous catheter, restricted patients’ movement to some extent.

“I have just finished surgery, I am afraid of touching the thoracic drainage tube and urinary catheter when I exercise in bed.” (P7, 68 years).

Three participants (20.0%) indicated that the change in therapeutic drugs may lead to altered symptoms and affect the exercise rehabilitation.

“In the last week, my blood concentration of tacrolimus was not well controlled (...). I have diarrhea for two days and I can’t exercise. I plan to go to the hospital.” (P10, 75 years).

Seven participants (46.67%) indicated that they suffered from a variety of complications, which caused a serious burden on exercise rehabilitation.

“I have hypertension and diabetes, and my physical condition is relatively poor. After exercising for a while, I will break out in a cold sweat and feel dizzy.” (P3, 69 years).

##### Insufficient exercise endurance (fatigue, frailty)

LTx recipients often experience muscle weakness, osteoporosis, and other negative consequences due to surgical stress, immunosuppressive medication usage, and impaired skeletal muscle oxidative capacity. All participants reported that feelings of fatigue and frailty diminished their exercise endurance.

“I still feel weak after LTx. I used to move things and do housework effortlessly, but now I feel a little chest tightness when walking, let alone other exercise.” (P2, 56 years).

#### Facilitators

##### Physical condition improved after transplantation

Ten participants (66.67%) confirmed that undertaking exercise rehabilitation notably enhanced their physical functions. A fascinating trend also emerged: in the initial year following LTx, a patient’s physical activity level typically decreased, but the trend would reverse thereafter, gradually rising to surpass the level prior to the transplantation.

“I persevered with cycle ergometer training for a week, and I noticed a significant increase in the strength of my lower limbs.” (P15, 61 years).

“When I initially began exercising, I felt a lack of strength in my body. But after a year of persistence, my limbs were slowly gaining strength. Now there is no problem in my daily life. I still insist on exercising every day and look forward to returning to my former state.” (P11, 45 years).

“After exercising for a while, I found that my physical condition was getting better and better. Now I have been insisting for more than a year. I really think that exercise is very important.” (P5, 55 years).

##### Reduce discomfort symptoms

Participants reported experiencing several benefits from exercise rehabilitation, such as the overall improvement of respiratory symptoms, enhanced activity ability, and increased physical comfort.

“After the operation, I still feel chest tightness. Under the guidance of healthcare providers, I insisted on exercising. Now I feel that my breathing is smooth and my limbs are strong.” (P8, 51 years).

### Psychological capability

#### Barriers

##### Lack of exercise-related knowledge (patient, healthcare providers)

Driven by the concept of accelerated rehabilitation, the hospitalization time of patients was obviously shortened. Nevertheless, six patients (40.0%) said that their knowledge about exercise during home rehabilitation was weak and their health education from professionals was limited.

“I know I’m going to start exercising. During my stay in the hospital, both doctors and nurses instructed me to exercise, but after I stayed at home, I was a little at a loss and couldn’t master the degree of exercise well.” (P8, 51 years).

##### Healthcare providers’ conflicting or vague advice

The majority of patients expressed their expectation of early exercise, but the health education provided by healthcare providers primarily centered around exercise rehabilitation programs, in which there was a lack of systematic guidance during the actual exercise sessions.

“I can understand the precautions explained by healthcare providers, but I always feel wrong when I actually do it. I still have difficulties in exercising myself, and I want the healthcare providers to guide me.” (P14, 57 years).

##### Negative life events after transplantation (risk perception)

All participants said that although the operation was successful, they were still worried about postoperative life and were afraid of complications such as infection and tumors. In particular, the outbreak of COVID-19 epidemic aggravated their worries. Then they chose to avoid possible risks, which reduced the compliance of exercise rehabilitation.

“I dare not move casually after the operation. I am worried that I will suddenly feel chest tightness and asthma after exercise. The feeling is particularly uncomfortable, and I still have a lingering fear.” (P7, 68 years).

#### Facilitators

##### Actively learn basic exercise knowledge

The proficiency with which patients understand exercise rehabilitation knowledge has a direct impact on their exercise behaviors. Some participants opined that actively seeking knowledge about exercise helped them to understand its value and benefits.

“After I learned how to exercise before the operation, I became more active after the operation.” (P4, 33 years).

##### Actively seek professional guidance

The importance of professional teams in bolstering patients’ trust and adherence towards exercise was highlighted. Participants agreed that clear guidance and education about rehabilitation from healthcare providers was direct and effective.

“Doctors and nurses gave me exercise rehabilitation instruction manuals, which I thought were particularly useful. The contents were very clear, so I just followed them.” (P15, 61 years).

##### Positive improvement of exercise behavior

Six participants (40.0%) stated that seeing positive results from exercising invigorated their motivation, with the improvement in exercise endurance reinforcing the formation of their beliefs about the efficacy of exercise.

“My family has specially equipped me with sports equipment. After a month of exercise, I have climbed four flights of stairs now, and I have no symptoms of chest tightness or asthma. I think I have made great progress.” (P6, 56 years).

### Physical opportunity

#### Barriers

##### Bad weather

Some participants expressed that despite their desire to exercise, they faced challenges in developing consistent exercise rehabilitation habits due to the constraints of their living conditions and the natural environment.

“It is too cold or too hot for me to go out for exercise.” (P9, 59 years).

##### The limitation of external environment (the surrounding environment lacks exercise facilities/equipment, facilities for exercise are inaccessible in the community and at home)

The external environment, including the availability of exercise locations and facilities, plays a pivotal role in shaping the exercise rehabilitation experience.

“There are no exercise facilities around my home. I need to go to other places for exercise by car. This is very difficult for me, and it is far away and expensive. I am also afraid of any accidents when I exercise.” (P6, 56 years).

##### Heavy financial burden

Due to the high cost of transplant operation and long-term use of immunosuppressants after operation, some participants complained of heavy economic burden.

“I used to have a job, but now I can’t work after the operation. The cost of the whole operation and postoperative treatment is relatively high. I am under great pressure and don’t want to exercise.” (P13, 49 years).

#### Facilitators

##### Accessibility and convenience of exercise

The accessibility and convenience of exercise resources in the living environment can easily stimulate participants’ desire for exercise.

“There is a square in our community, and we have a square dance every night. I think it is very useful for my health.” (P8, 51 years).

### Social opportunity

#### Barriers

##### Lack of social support

Social support encompasses the encouragement and assistance provided by family, friends, and colleagues, among others. However, it was noted that some participants encountered limited levels of support from these people.

“My family and friends think that exercise is not effective and do not support me to continue to exercise (...).” (P4, 33 years).

##### Insufficient support from healthcare providers

Nearly all participants expressed a desire for healthcare providers to provide them with more comprehensive health guidance about their diseases. However, healthcare providers often failed to effectively communicate with the participants and meet their knowledge needs.

“Doctors don’t communicate with me much; I especially want to know more about LTx disease counseling, home care, follow-up contact(...).” (P14, 57 years).

##### Negative social effect

Most of the participants talked about the misunderstood perceptions of LTx in society. The pressure of external public opinion would lead to a sense of shame, forcing them to flinch from social activities.

“I seldom go out to exercise (...). In our countryside, when it comes to “transplantation“, everyone thinks that this disease may be contagious. I am afraid that others will forsake me.” (P8, 51 years).

#### Facilitators

##### Peer support

Peer support plays a crucial role in boosting patients’ confidence to overcome challenges in exercise rehabilitation. Participants expressed that sharing successful experiences with fellow LTx patients helped strengthen their belief in the efficacy of exercise rehabilitation.

“I joined a WeChat group consisting of LTx patients. We made an appointment to exercise and connected the dragon every day. I feel very good.” (P11, 45 years).

“I am especially willing to participate in activities with fellow patients because we share a common topic. We also set up a WeChat group to often talk about how to exercise after transplantation.” (P5, 55 years).

##### Professional support from healthcare providers

Participants acknowledged their dependence on healthcare providers post-illness, and they found that receiving care and support from them greatly enhanced their motivation for exercise.

“The healthcare providers regularly contacted me after my discharge, providing guidance for home-based exercise and boosting my confidence to continue exercising.” (P15, 61 years).

### Automatic motivation

#### Barriers

##### Ambivalence in decision making

Participants have limited opportunities to participate in decision making because of the special decision-making situation.

“Now I don’t know what to do about many things. For example, I want to take an exercise after the operation, but my family thinks I should rest, and I’m afraid I won’t get good results after I insist. I don’t know whether I should insist on exercising or not, and my mind is very chaotic.” (P14, 57 years).

##### Anxiety about impaired health

Some participants have health-related concerns about participating in social activities. They are afraid that it may damage their health, so they avoid social activities.

“Affected by the COVID-19 epidemic, many friends wanted to visit me, but I didn’t let them come. My immunity was low after the operation, and I was afraid it would affect me.” (P1, 24 years).

##### Worries about the outcomes of LTx (infection, graft loss)

The treatment after LTx is complicated, and symptoms such as infection and graft loss are obstacles to exercise rehabilitation. All participants said that they were worried about the transplant outcomes during exercise.

“I am particularly worried about infection when I exercise. I don’t know how to avoid infection. I only know cleaning and taking anti-infective drugs on time”. (P5, 55 years).

“If other problems damage pulmonary function and eventually lead to graft loss, I will collapse. So I dare not exercise, for fear that being too tired will affect pulmonary function.” (P12, 50 years).

##### Discomfort experience brought by exercise (asthma, chest stress)

Six participants (40.0%) have uncertainty and doubts about the safety of exercise rehabilitation behavior after experiencing bad symptoms.

“I didn’t pay attention to breathing and blood oxygen saturation before exercise (...). As a result, I had asthma and decreased blood oxygen saturation after exercise.” (P13, 49 years).

#### Facilitators

##### Perceive the significance of exercise rehabilitation

Four participants (26.67%) emphasized that undergoing LTx not only improves their organ health but also grants them a new lease on life. They consider it a unique challenge and opportunity, with some expressing gratitude and contentment towards their new life.

“After this major operation, I realized that people should learn to be grateful and satisfied. I must exercise diligently and make the most of my second chance at life.” (P8, 51 years).

##### Optimistic exercise attitude

Following the traumatic experience of LTx, five participants (33.33%) reported a positive transformation in their overall outlook on life.

“I feel my breathing suddenly becoming unobstructed. This feeling is really wonderful. I want to exercise well, improve my physique and live a happy life.” (P11, 45 years).

##### Adapting to LTx and reinventing oneself

Patients encounter various emotional stresses throughout the LTx process. However, after transplantation, some participants said they have become more open-minded and optimistic due to their regained health.

“After LTx, my heart suddenly calmed down and I felt suddenly enlightened. I believe this is the best arrangement for me now.” (P15, 61 years).

### Reflective motivation

#### Barriers

##### Excessive self-expectation

Participants in a stable period or home rehabilitation period may be frustrated due to insufficient understanding of rehabilitation goals and lower rehabilitation effect than self-expectation.

“I have been exercising for three months. I thought my physical fitness would be improved obviously, but in fact I am still weak and can’t lift heavy objects.” (P4, 33 years).

##### Lack of exercise confidence

Diverse beliefs and levels of confidence regarding exercise rehabilitation can result in varying coping behaviors. Eight participants (53.33%) expressed doubt about their ability to return to their pre-illness state, leading them to take conservative or evasive actions.

“Although I have finished the operation, I still feel very sick, and I don’t have the strength to exercise. I’m also afraid that there will be other problems in case (...). I’m not sure.” (P2, 56 years).

##### Not be included in the priority target

Target priority affects participants’ exercise rehabilitation. Exercise is postponed when patients believe that other postoperative treatments are more effective than exercise.

“I think the most important thing after transplantation is to actively cooperate with postoperative treatment, such as taking immunosuppressive drugs, so exercise can be postponed.” (P7, 68 years).

##### Lack of motivation (lack of interest in exercise rehabilitation, dislike of exercise)

Motivation is the internal driving force that affects patients’ exercise rehabilitation behavior. Some participants in the early treatment stage paid more attention to the current illness and treatment effect, and could not perceive the long-term benefits brought by exercise.

“My friend called me to participate in group exercise, (...). I don’t think it makes any sense, and I don’t like it. I don’t have great expectations for postoperative life.” (P4, 33 years).

#### Facilitators

##### High self-efficacy

Participants’ self-efficacy can improve their problem-solving ability, enabling them to overcome fatigue symptoms and establish exercise behavior.

“After the operation, I felt very tired when I started to exercise. But I think I have finished the transplant operation, so what else can I not overcome?” (P1, 24 years).

##### Priority of moving target

The disease disrupted the patient’s original life rhythm and planning. Nevertheless, after the operation, six participants (40.0%) expressed that they had reevaluated and rearranged the priorities in their post-operative lives, with exercise becoming a primary focus for their future.

“I used to think that work was of utmost important, but now exercise to keep healthy is the first priority. I adhere to my exercise plan every day, gradually increasing it from 2 days a week, 30 minutes a day, to 5 days a week. I believe exercise has become an integral part of my life, and I aspire to return to normal life as soon as possible.” (P8, 51 years).

##### Enhanced motivation for exercise rehabilitation (positive psychological experiences)

Seven participants (46.67%) reported that exercise rehabilitation brought about positive psychological experiences, which, in turn, boosted their motivation to continue exercising.

“I felt uncomfortable at the beginning of exercise, but I found that my body is becoming healthier and I feel full of energy every day.” (P15, 61 years).

## Discussion

This study utilized the COM-B theory and TDF to explore the barriers to and facilitators of exercise rehabilitation among adult LTx patients. Data from 6 major themes and 12 domains were collected, and perceived barriers or facilitators were categorized into 9 domains. The study identified significant barriers to exercise rehabilitation, such as physical limitations, insufficient exercise endurance, lack of knowledge, motivation, among others. Key facilitators included motivation, self-efficacy, perceived significance of exercise rehabilitation, and social support and so on. These findings are consistent with those of Gustaw et al. [12]. Moreover, this study addresses unique challenges that LTx patients face when participating in exercise rehabilitation, such as pain, pipelines, drug side effects, perceived risks associated with exercise after transplantation, decision-making ambivalence, and discomfort during exercise. The results of this study complement existing literature and may serve as valuable references for the development and implementation of exercise rehabilitation intervention strategies for adult LTx patients in the future.

In the COM-B model [25], various factors influence behavior, including ability factors (psychological and physiological abilities, such as physical skills, knowledge, and emotions), opportunity factors (physical and social environments, such as environmental resources and social influences), and motivational factors (such as goals and self-efficacy). Besides, the TDF, proposed by Michie et al. [26], integrates multiple behavior-change theories and helps us identify the specific factors that influence target behavior in a more concrete and detailed manner. Our findings from this qualitative study aligned closely with the COM-B model. We identified the barriers and facilitators of exercise rehabilitation and found that individual behavior change can only be motivated and guided when individuals possess the necessary ability, opportunity, and motivation. By combining the TDF, our comprehensive analysis of the influencing factors of behavior can offer a clear and detailed practical framework for the behavioral interventions, ensuring their alignment with behavioral mechanisms [33, 34].

Based on the COM-B theory and TDF, this study revealed that physical ability was the most prominent factor hindering exercise rehabilitation. This primarily referred to insufficient exercise capacity or muscle strength, as well as the impact of pain or comorbidities. Additionally, comorbidities and medication side effects were identified as secondary barriers. A study [35] of solid organ transplant recipients indicated that physical limitations, low energy levels and muscle weakness were important barriers, which was consistent with our results. Diminished physical ability in LTx recipients may be the consequence of a combination of preoperative pulmonary disease, chronic disorders, and postoperative factors, for instance, postoperative muscle limitations in the lower limbs, upper limbs, and trunk, as well as early decreases in muscle mass and strength, type 1 fiber ratio, calcium uptake and release, mitochondrial enzyme activity, muscle pH, and impaired peripheral muscle oxidative capacity [36, 37].

The study also shed light on several psychological factors that hinder the participation of adult LTx patients in exercise rehabilitation. These factors include lack of exercise knowledge, conflicting or vague advice from healthcare providers, and negative life events (perceived risks) following transplantation. Furthermore, exercise-related knowledge mainly comes from patients’ self-study and healthcare providers, which is crucial to exercise behavior. Healthcare providers can provide patients with extensive exercise-related knowledge and guidance, such as the benefits of active exercise to health, as well as where, when and how to exercise, to help them overcome barriers. Yet, previous research has not highlighted the issue of healthcare providers delivering confusing or contradictory advice as a potential obstacle [35, 38, 39]. Our findings underscore the importance of active knowledge seeking, professional guidance, and proactive efforts to improve exercise behavior in enhancing psychological well-being.

Participants in this study also reported physical opportunity-related factors. They mentioned that the lack of exercise facilities/equipment and affordable exercise rehabilitation resources in their communities and families, as well as the economic burden, discouraged their participation in exercise. Several themes identified in this study aligned with the barriers (such as weather, environmental constraints, and economic factors) and facilitators (like social support and availability of resources) for elderly patients with debilitating cancer to engage in exercise rehabilitation before surgery [40]. Therefore, it is advisable to create an environment that promotes physical activities and provide opportunities for patients to engage in sports.

This study revealed that social opportunity-related barriers mainly included a lack of social support, insufficient support from healthcare providers and negative social influence, while facilitators included peer support and professional support from healthcare providers. A study by Wietlisbach et al. [41] indicated that patients who had undergone LTx for less than 3 years had higher needs for improved quality of life, achieving personal goals, support from others/family, and suggestions from healthcare providers than those who had undergone transplantation for 10 years, and they were less confident in their new organ. It was found that face-to-face educational seminars, encompassing expert lectures on exercise and exercise training specifically tailored to organ transplant patients, can improve PA levels [42]. This suggests that future efforts should incorporate planned and systematic education and training for multidisciplinary professionals such as healthcare providers, physiotherapists, or kinesiologists. This would enable them to develop appropriate plans and goals based on the patient’s time since transplantation, personal interests, and individual needs [43]. Furthermore, Wickerson et al. [44] found high levels of utilization and satisfaction with tele-rehabilitation among LTx patients. This indicates that internet platforms and digital health tools [21, 45] (such as artificial intelligence, digital medicine, big data, etc.) can be leveraged to implement remote pulmonary rehabilitation interventions and care plans [46] for LTx patients.

The study also indicated that barriers related to spontaneous motivation included decision-making ambivalence, anxiety about health impairment, fear of LTx outcomes, and exercise-related discomfort. Facilitators included the perceived significance of exercise rehabilitation, positive attitudes toward exercise, and adaptation to LTx. These findings were consistent with previous investigations into PA disorders in renal transplant patients, which identified factors such as fear of harming transplanted organs and physical insecurity [47]. In the future, it is essential to increase awareness of the benefits of exercise and the consequences of not exercising in order to stimulate patients’ sense of responsibility towards their new organs. Encouraging family and friends to accompany patients in participating in recreational exercises and group activities may also prove beneficial [48].

The study unveiled various barriers related to reflective motivation, including excessive self-expectations, a lack of exercise confidence, not being prioritized as a target, and a lack of motivation. On the other hand, facilitators of reflective motivation included a high level of self-efficacy, priority of moving target, and exercise motivation. These findings aligned with a previous study on adult LTx recipients with cystic fibrosis who also faced barriers such as insufficient energy, competing commitments, and lack of time for PA [41]. According to behavioral science theory, motivation plays a crucial role in engaging in and adhering to PA [49]. Previous research also found that individuals with higher levels of motivation tend to exhibit higher self-efficacy and engage in more PA [50]. As recovery from disabilities progresses, exercise and participation tend to increase [51]. Interventions based on social cognitive theory, self-management strategies, and motivational interviewing techniques have been designed to address potential barriers to PA and enhance motivation [52]. Cognitive-behavioral therapy aids patients in acquiring coping skills for managing psychological stress (such as relaxation training and cognitive reorganization) and improving sports cognition and self-management abilities. This approach is worth considering. Additionally, tailoring exercise plans based on behavior change theory, patients’ abilities, and preferences can be effective in jointly developing action plans and goals with patients to stimulate their intrinsic motivation. Verbal persuasion, peer models, and accurate interpretation of exercise-related physical cues can also be utilized to improve self-efficacy [53].

Despite the strengths of this study, it also has certain limitations. Firstly, almost all participants were male, which may have missed potential gender differences in exercise adherence cognition. Secondly, there may be a sample selection bias as the participants were recruited from a single hospital, and their age ranged from 24 to 75 years old, which could influence the generalizability of the findings. Thirdly, these interviews were conducted at various time points ranging from 0.1 to 43 months after surgery, and participants may have changed their perspectives and exercise behaviors as time went by. Therefore, it remains unclear whether these perspectives remain consistent over time. Future research should aim to conduct longitudinal interviews to understand any potential changes in long-term exercise adherence trajectories.

## Conclusions

In conclusion, this study shed light on the challenges in maintaining exercise rehabilitation in adult LTx patients, and identified the main barriers and facilitators. Overall, patients demonstrated a positive attitude towards exercise rehabilitation and expressed a desire for support, indicating the need for active behavior change strategies to promote their participation. The application of the COM-B model and TDF in this study yielded comprehensive and systematic results with significant clinical implications. It not only identified the factors influencing patients’ behavior but also provided a direct basis for the development of patient-centered behavior change strategies for exercise rehabilitation. Further research is warranted to explore the use of professional support, pulmonary rehabilitation training, behavior change technologies, and digital health tools, which will be crucial for strengthening the evidence base on exercise efficacy and effectiveness.

## Data Availability

Data are available from the author HY upon reasonable request to the authors.
